# Infarcted fibroadenoma of the breast: report of two new cases with review of the literature

**DOI:** 10.1186/1746-1596-8-38

**Published:** 2013-02-27

**Authors:** Faruk Skenderi, Fikreta Krakonja, Semir Vranic

**Affiliations:** 1 Department of Pathology, Clinical Center of the University of Sarajevo, Bolnička 25, Sarajevo, BA-71000, Bosnia and Herzegovina; 2 Department of Radiology, Clinical Center of the University of Sarajevo, Sarajevo, Bosnia and Herzegovina

**Keywords:** Breast tumors, Benign tumors, Fibroadenoma, Infarction, Necrosis

## Abstract

**Introduction:**

Fibroadenomas are the most common benign breast tumors in young women. Infarction is rarely observed in fibroadenomas and when present, it is usually associated with pregnancy or lactation. Infarction can exceptionally occur as a complication of previous fine-needle aspiration biopsy or during lactation and pregnancy.

**Materials and methods:**

Retrospective review of 650 cases of fibroadenomas diagnosed at our institution during the 8-years period identified two cases of fibroadenomas with infarction (rate ~0.3%).

**Results:**

Two partially infarcted fibroadenomas were diagnosed on core biopsy and frozen section in an adolescent girl (13 years old) and in a young woman (25 years old), respectively. No preceding fine-needle aspiration biopsy was performed in these cases, nor were the patients pregnant or lactating at the time of the diagnosis.

**Conclusion:**

Spontaneous infarction within fibroadenoma is a rare phenomenon in younger patients. The presence of necrosis on core biopsy or frozen section should be cautiously interpreted and is not a sign of malignancy.

**Virtual Slides:**

The virtual slide(s) for this article can be found here: http://www.diagnosticpathology.diagnomx.eu/vs/1556060549847356

## Introduction

Fibroadenomas are the most common benign neoplasms of the breast usually affecting adolescents and young women [1,2]. Infarction in benign breast lesions is rare and may occur in various conditions, including fibroadenomas [1,3]. Infarction within a fibroadenoma was first described by Delarue and Redon in 1949 [4] and usually affects young women during pregnancy or lactation, but may occur at any age following fine-needle aspiration biopsy (FNA) [1,5-8].

Clinically, fibroadenoma typically presents as a palpable mass and may occasionally be mistaken for inflammatory lesions due to pain and tenderness or for malignancy due to hardness, fixation to the surrounding tissue or bloody nipple discharge [2,9,10].

Spontaneous infarction of fibroadenomas not related to previously mentioned causes occurs exceptionally and only a few cases are described in the available literature [2,7,9,11-14].

Herein, we describe a review of fibroadenomas of the breast with two new cases of spontaneous infarction, unrelated to any known risk factor.

## Materials and methods

We did a retrospective search of our database for fibroadenomas of the breast that were diagnosed at our department during the 8 years period (2005–2012). Two cases of fibroadenoma with spontaneous infarction were identified. Paraffin-embedded tissue blocks and hematoxylin and eosin (H&E) slides were retrieved from the pathology archive and retrospectively reviewed (F.S. and S.V.).

In a case #1 immunohistochemical staining against estrogen receptor (ER, clone: 1D5, Dako, Glostrup, Denmark) and progesterone receptor (PR, clone: PgR636, DAKO, Glostrup, Denmark) was performed.

Clinical history was available for both cases along with radiologic images (ultrasound and magnetic resonance imaging [MRI]).

## Results

Our search revealed 650 fibroadenomas during the period 2005–2012. Infarcted fibroadenomas were diagnosed in two patients (rate 0.3%): an adolescent girl (13 years old, Case #1) and in a young woman (25 years old, Case #2). Both patients had no previous history of pregnancy, lactation or previous FNA. No hormonal disturbances were present in the patients. Postoperative course was uneventful in both patients.

### Case 1

**Figure 1:**
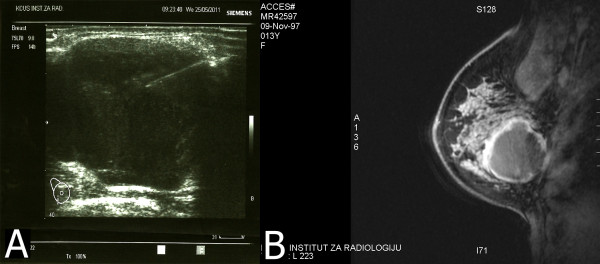
Ultrasound examination (A) and MRI (B) of the breast (Case #1) revealed a circumscribed, hypoechoic mass, measuring 40 mm.

A 13-year-old pubertal girl presented with a rapidly growing, mobile and painful mass in the right breast. Radiologic findings (ultrasound and MRI) indicated the presence of a well-circumscribed tumor of the right breast consistent with a juvenile fibroadenoma (BI-RADS Category 2) (Figure 1A-B).

**Figure 2:**
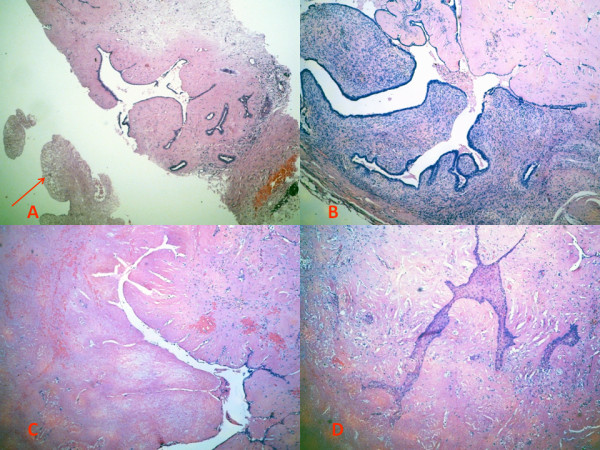
(A): Hematoxylin & Eosin (H&E) slide of a core biopsy showing a classical fibroadenoma with an area of necrosis (arrow) (5x); (B): An excision biopsy revealing a fibroadenoma with a focal morphologic features of benign phyllodes tumor (H&E, 10x); (C): A zone of necrosis (H&E, 10x); (D): Squamous metaplasia, observed in close proximity of necrotic foci, reminiscent of the so-called necrotizing syringometaplasia in the skin or necrotizing sialometaplasia in salivary glands (H&E, 10x).

Radiologist performed a core needle biopsy that revealed a fibroepithelial lesion exhibiting focal necrosis but without malignant cells (classified as B3 lesion) which led to the immediate excision biopsy, performed a week later (Figure 2A).

Grossly, the tumor was an encapsulated, soft yellow, measuring 40×35×25 mm with foci of hemorrhage and necrosis. The entire tumor was submitted for microscopic evaluation. Microscopic findings were consistent with partially infarcted benign fibroadenoma with focal increased stromal cellularity resembling that of benign phyllodes tumor (Figure 2B-C). Squamous metaplasia, observed in close proximity to the necrotic foci, was present and was reminiscent of the so-called necrotizing syringometaplasia in the skin or necrotizing sialometaplasia in salivary glands (Figure 2D).

In Case #1, both epithelial and stromal cells were focally positive for estrogen receptor (ER) and progesterone receptor (PR) (~20% of the cells with weak to moderate nuclear intensity).

### Case 2

A 25-year-old gravida 0, para 0 woman presented to a breast radiologist with a short history of a rapidly growing palpable and painful tumor in the left breast. Clinical and radiologic findings (ultrasound) were suggestive of juvenile fibroadenoma or intraductal papilloma (BI-RADS Category 2) and the patient was referred to the breast surgeon. The excision biopsy was performed and intraoperative frozen section consultation was obtained which read as a benign fibroepithelial tumor with focal intratumoral hemorrhage without signs of malignancy.

**Figure 3:**
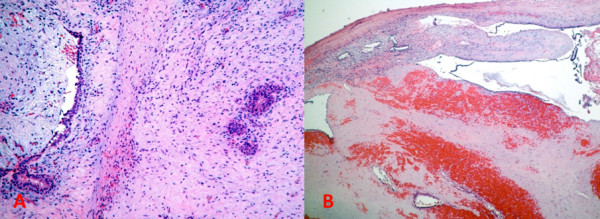
(A): A classical cellular (juvenile) fibroadenoma (H&E, 10x); (B): An area with hemorrhage and necrosis (H&E, 5x).

Gross examination revealed a nodular, circumscribed tumor, measuring 25×20×17 mm that was soft, yellow with peripheral areas of hemorrhage. The entire tumor was submitted for histopathologic evaluation which revealed juvenile (cellular) fibroadenoma with foci of hemorrhage and necrosis (Figure 3A-B). No evidence of malignancy was found.

## Discussion

Fibroadenomas are the most common benign tumors of the breast that usually affect premenopausal women but may occur at any age. Diagnosis of fibroadenoma rarely poses a diagnostic dilemma, even on core biopsy, FNA or frozen section. We presented here two rapidly growing infarcted fibroadenomas that were causing pain. Infarction within fibroadenoma is a very rare event and the frequency of infarction in our study is in line with a study of Haagensen [15] who found only five infarcted cases among 1,000 reviewed breast fibroadenomas (rate: 0.5%). Another two studies based on the series of fibroadenomas in West African women revealed a slightly higher incidence of spontaneous infarction (0.9%) [16,17]. The highest frequency (3.6%) was reported in a recent study of Al-Atrooshi [18].

The presence of infarction and necrosis are usually worrisome signs in breast pathology although spontaneous infarction can be seen in variety of benign breast lesions including fibroadenoma, phyllodes tumor, lactating adenoma, and intraductal papilloma [3,19-23]. Spontaneous infraction in fibroadenomas is a rare phenomenon and usually associated with pregnancy, lactation or a recent FNA [5-7]. Exceptionally, spontaneous infarction may affect multiple fibroadenomas in the same patient [24]. Infarction may also be associated with the use of oral contraceptives [25]. Rarely, it can be seen in young patients without any associated risk factors, as illustrated in our two cases. The cause and mechanism of infarction are largely unknown. One of the possible explanations is that infarction represents a spectrum of regressive changes that also may include calcification and hyalinization, both of which are much more commonly seen in fibroadenomas [26]. Newman et al. [27] also found thrombo-oclussive vascular changes as a possible cause of infarction within fibroadenomas.

In our first case, a focus of necrosis was seen on core biopsy which prompted excision biopsy while in the second case clinical suspicion for malignancy prompted an intra-operative frozen section consultation which also revealed the presence of intratumoral hemorrhage and necrosis. A meticulous histopathologic evaluation of the entire tumors, however, revealed no signs of malignancy despite the presence of necrosis. Of note, case #1 also showed areas of squamous metaplasia resembling so-called necrotizing syringometaplasia in the skin or sialometaplasia in salivary glands. This phenomenon has already been described in infarcted breast fibroadenomas [28] and can also be seen in other benign breast lesions in a close proximity to the area of infarction (e.g. intraductal papilloma, [20]).

Fibroadenomas, particularly in older women, may be affected by various proliferative changes including malignant epithelial lesions [29,30]. The most frequent are in situ carcinomas (both ductal and lobular), and their invasive counterparts [30-32]. Exceptionally, sarcomas may also develop within fibroadenomas (e.g. angiosarcoma, osteosarcoma) [33,34]. Little is known about the molecular mechanisms that drive the development and prog ression of malignant tumors within fibroadenomas. Comparative studies that analyzed various molecular markers in fibroadenomas and breast carcinomas failed however to identify the potential drivers [35,36]. However, a clonality study of Kuijper et al. [37] indicated that fibroadenomas possessed a potential to progress in an epithelial direction (carcinoma) or in a stromal direction (phyllodes tumor).

We conclude that partial spontaneous infarction is a rare event in breast fibroadenomas and may not be associated with any known risk factor. The presence of necrosis on core biopsy or intra-operative frozen section should be cautiously interpreted and is not itself a sign of malignancy.

## Consent

The case reports were shared with the local ethical committee whose policy is not to review case reports.

## Competing interests

The authors declare no conflict of interest.

## Authors’ contributions

SV and FK have been directly involved in diagnosis and interpretation of patient’s diagnosis. FS and SV conceived the study design. All authors wrote and approved the final manuscript.

## References

[B1] Rosen PP Rosen’s breast pathology 2009 3 Lippincott Williams & Wilkins

[B2] Liu H Yeh ML Lin KJ Huang CK Hung CM Chen YS Bloody nipple discharge in an adolescent girl: unusual presentation of juvenile fibroadenoma Pediatr Neonatol 2010 51 190 192 10.1016/S1875-9572(10)60036-8 20675246

[B3] Fratamico FC Eusebi V Infarct in benign breast diseases. Description of 4 new cases Pathologica 1988 80 433 442 3072514

[B4] Majmudar B Rosales-Quintana S Infarction of breast fibroadenomas during pregnancy JAMA 1975 231 963 964 10.1001/jama.1975.03240210043017 1173104

[B5] Vargas MP Merino MJ Infarcted myxoid fibroadenoma following fine-needle aspiration Arch Pathol Lab Med 1996 120 1069 1071 12049112

[B6] Pinto RG Couto F Mandreker S Infarction after fine needle aspiration. A report of four cases Acta Cytol 1996 40 739 741 10.1159/000333949 8693896

[B7] Ichihara S Matsuyama T Kubo K Tamura Z Aoyama H Infarction of breast fibroadenoma in a postmenopausal woman Pathol Int 1994 44 398 400 8044310 10.1111/j.1440-1827.1994.tb02941.x

[B8] Lucey JJ Spontaneous infarction of the breast J Clin Pathol 1975 28 937 943 10.1136/jcp.28.12.937 1206116 PMC475908

[B9] Oh YJ Choi SH Chung SY Yang I Woo JY Lee MJ Spontaneously infarcted fibroadenoma mimicking breast cancer J Ultrasound Med 2009 28 1421 1423 19778895 10.7863/jum.2009.28.10.1421

[B10] Deshpande KM Deshpande AH Raut WK Lele VR Bobhate SK Diagnostic difficulties in spontaneous infarction of a fibroadenoma in an adolescent: case report Diagn Cytopathol 2002 26 26 28 10.1002/dc.10029 11782083

[B11] Fowler CL Spontaneous infarction of fibroadenoma in an adolescent girl Pediatr Radiol 2004 34 988 990 10.1007/s00247-004-1250-4 15368085

[B12] Toy H Esen HH Sonmez FC Kucukkartallar T Spontaneous Infarction in a Fibroadenoma of the Breast Breast Care (Basel) 2011 6 54 55 10.1159/000324047 21547027 PMC3083272

[B13] Hsu S Hsieh H Hsu G Lee H Chen K Yu J Spontaneous Infarction of a Fibroadenoma of the Breast in a 12-Year-Old Girl Journal of medical sciences Taipei 2005 25 313

[B14] Meerkotter D Andronikou S Unusual presentation and inconclusive biopsy render fibroadenoma in two young females a diagnostic dilemma: case report SA Journal of Radiology 2009 13 62 65

[B15] Haagensen CD Diseases of the Breast 1986 Saunders

[B16] Jayasinghe Y Simmons PS Fibroadenomas in adolescence Curr Opin Obstet Gynecol 2009 21 402 406 10.1097/GCO.0b013e32832fa06b 19606032

[B17] Onuigbo W Breast fibroadenoma in teenage females Turk J Pediatr 2003 45 326 328 14768798

[B18] Al-Atrooshi SA Fibroepithelial tumors of female breast: a review of 250 cases of fibroadenomas and phylloides tumors The Iraqi Postgraduate Medical Journal 2012 11 140 145

[B19] Verslegers I Tjalma W Van Goethem M Colpaert C Biltjes I De Schepper AM Parizel PM Massive infarction of a recurrent phyllodes tumor of the breast: MRI-findings JBR-BTR 2004 87 21 22 15055329

[B20] Flint A Oberman HA Infarction and squamous metaplasia of intraductal papilloma: a benign breast lesion that may simulate carcinoma Hum Pathol 1984 15 764 767 10.1016/S0046-8177(84)80168-9 6745916

[B21] Behrndt VS Barbakoff D Askin FB Brem RF Infarcted lactating adenoma presenting as a rapidly enlarging breast mass AJR Am J Roentgenol 1999 173 933 935 10.2214/ajr.173.4.10511151 10511151

[B22] Baker TP Lenert JT Parker J Kemp B Kushwaha A Evans G Hunt KK Lactating adenoma: a diagnosis of exclusion Breast J 2001 7 354 357 10.1046/j.1524-4741.2001.20075.x 11906446

[B23] Okada K Suzuki Y Saito Y Umemura S Tokuda Y Two cases of ductal adenoma of the breast Breast Cancer 2006 13 354 359 10.2325/jbcs.13.354 17146162

[B24] Akdur NC Gozel S Donmez M Ustun H Spontaneous infarction of multiple fibroadenoma in a postlactational woman: case report Turkiye Klinikleri Journal of Medical Sciences 2012 32 1429 1432 10.5336/medsci.2010-22098

[B25] Tavassoli FA Pathology of The Breast 1999 McGraw-Hill

[B26] Greenberg R Skornick Y Kaplan O Management of breast fibroadenomas J Gen Intern Med 1998 13 640 645 10.1046/j.1525-1497.1998.cr188.x 9754521 PMC1497021

[B27] Newman J Kahn LB Infarction of fibro-adenoma of the breast Br J Surg 1973 60 738 740 10.1002/bjs.1800600921 4741192

[B28] Pandit AA Deshpande RB Infarction of fibroadenoma with squamous metaplasia Indian J Cancer 1985 22 271 273 3843092

[B29] Tissier F De Roquancourt A Astier B Espie M Clot P Marty M Janin A Carcinoma arising within mammary fibroadenomas. A study of six patients Ann Pathol 2000 20 110 114 10740004

[B30] Goldman RL Friedman NB Carcioma of the breast arising in fibroadenomas, with emphasis on lobular carcinoma. A clinicopathologic study Cancer 1969 23 544 550 10.1002/1097-0142(196903)23:3<544::AID-CNCR2820230305>3.0.CO;2-F 5766498

[B31] Diaz NM Palmer JO McDivitt RW Carcinoma arising within fibroadenomas of the breast. A clinicopathologic study of 105 patients Am J Clin Pathol 1991 95 614 622 1850948 10.1093/ajcp/95.5.614

[B32] Cole-Beuglet C Soriano RZ Kurtz AB Goldberg BB Fibroadenoma of the breast: sonomammography correlated with pathology in 122 patients AJR Am J Roentgenol 1983 140 369 375 10.2214/ajr.140.2.369 6600356

[B33] Babarovic E Zamolo G Mustac E Strcic M High grade angiosarcoma arising in fibroadenoma Diagn Pathol 2011 6 125 10.1186/1746-1596-6-125 22185665 PMC3284406

[B34] Killick SB McCann BG Osteosarcoma of the breast associated with fibroadenoma Clin Oncol (R Coll Radiol) 1995 7 132 133 10.1016/S0936-6555(05)80817-9 7619763

[B35] Bal A Joshi K Logasundaram R Radotra BD Singh R Expression of nm23 in the spectrum of pre-invasive, invasive and metastatic breast lesions Diagn Pathol 2008 3 23 10.1186/1746-1596-3-23 18510781 PMC2423356

[B36] Xu X Jin H Liu Y Liu L Wu Q Guo Y Yu L Liu Z Zhang T Zhang X Dong X Quan C The expression patterns and correlations of claudin-6, methy-CpG binding protein 2, DNA methyltransferase 1, histone deacetylase 1, acetyl-histone H3 and acetyl-histone H4 and their clinicopathological significance in breast invasive ductal carcinomas Diagn Pathol 2012 7 33 10.1186/1746-1596-7-33 22455563 PMC3349567

[B37] Kuijper A Buerger H Simon R Schaefer KL Croonen A Boecker W van der Wall E van Diest PJ Analysis of the progression of fibroepithelial tumours of the breast by PCR-based clonality assay J Pathol 2002 197 575 581 10.1002/path.1161 12210075

